# Vpx enhances innate immune responses independently of SAMHD1 during HIV-1 infection

**DOI:** 10.1186/s12977-021-00548-2

**Published:** 2021-02-09

**Authors:** Oya Cingöz, Nicolas D. Arnow, Mireia Puig Torrents, Norbert Bannert

**Affiliations:** Department of Infectious Diseases HIV and Other Retroviruses, Robert Koch Institute, Berlin, Germany

**Keywords:** Infection, Innate immunity, Interferon, MDM, Macrophage, THP-1, ISG, ISRE

## Abstract

**Background:**

The genomes of HIV-2 and some SIV strains contain the accessory gene *vpx*, which carries out several functions during infection, including the downregulation of SAMHD1. Vpx is also commonly used in experiments to increase HIV-1 infection efficiency in myeloid cells, particularly in studies that investigate the activation of antiviral pathways. However, the potential effects of Vpx on cellular innate immune signaling is not completely understood. We investigated whether and how Vpx affects ISG responses in monocytic cell lines and MDMs during HIV-1 infection.

**Results:**

HIV-1 infection at excessively high virus doses can induce ISG activation, although at the expense of high levels of cell death. At equal infection levels, the ISG response is potentiated by the presence of Vpx and requires the initiation of reverse transcription. The interaction of Vpx with the DCAF1 adaptor protein is important for the enhanced response, implicating Vpx-mediated degradation of a host factor. Cells lacking SAMHD1 show similarly augmented responses, suggesting an effect that is independent of SAMHD1 degradation. Overcoming SAMHD1 restriction in MDMs to reach equal infection levels with viruses containing and lacking Vpx reveals a novel function of Vpx in elevating innate immune responses.

**Conclusions:**

Vpx likely has as yet undefined roles in infected cells. Our results demonstrate that Vpx enhances ISG responses in myeloid cell lines and primary cells independently of its ability to degrade SAMHD1. These findings have implications for innate immunity studies in myeloid cells that use Vpx delivery with HIV-1 infection.

## Background

Organisms are equipped with protective mechanisms against pathogenic invasions, for which interferons (IFNs) are critical regulators. For many viral pathogens, a functional innate immune system is essential for combatting infection both in the initial stages following virus encounter and for mounting highly specific adaptive immune responses at later stages of infection. At the cellular level, the coexistence of viruses with their hosts for millions of years have resulted in the evolution of multiple, robust, and sometimes overlapping cellular pathways that detect and respond to viral infections. It is therefore not surprising that many viruses, including human and simian immunodeficiency viruses (HIV and SIV), have also evolved strategies that specifically counteract these pathways and modify host responses.

The principal target of HIV-1 is CD4+ T-cells; however, dendritic cells, macrophages and natural killer cells are also activated during acute infection (reviewed in [1, 2]). IFNs and other proinflammatory cytokines produced during the acute phase help recruit additional target cells to the sites of replication and facilitate viral spread. In the later stages of infection macrophage-tropic viral isolates can arise, although rarely (reviewed in [3]), which replicate efficiently in the central nervous system and are often implicated in HIV-associated neurocognitive disorders. Thus, many different cell types are involved in the response to HIV-1 infection and it is important to understand how HIV-1 interacts with the innate immune system in these cells.

Complex retroviruses such as HIV and SIVs harbor multiple accessory genes that carry out various functions in infected cells, from counteracting specific restriction and resistance factors to cell cycle regulation, from transcriptional modulation of viral and host genes to alterations in cellular trafficking pathways, among others (reviewed in [4–6]). Vpx is an accessory gene that is absent in HIV-1, but present in several SIV strains as well as HIV-2, which allows efficient infection of myeloid cells and resting CD4+ T-cells [7–9]. Vpx interacts with the DCAF1 adaptor to associate with the CUL4-DDB1 ubiquitin ligase complex and target select host proteins for proteasomal degradation (reviewed in [6, 10]). The most prominently studied host targets of Vpx thus far are SAMHD1, which depletes cellular dNTP pools thereby preventing reverse transcription [11], and TASOR (FAM208A), which silences both unintegrated [12] and integrated, latent retroviral DNA [13, 14] as part of the HuSH complex [15]. As HIV-1 strains do not contain Vpx, the necessity of counteracting these factors has been brought into question (reviewed in [5]). However, SAMHD1 antagonism by Vpr is maintained during natural SIV infection in African green monkeys [16], suggesting a significant evolutionary pressure on viral fitness exerted by this host factor in vivo*.*

Many studies investigating innate immune responses against retroviruses utilize Vpx delivery to achieve high enough infection levels in myeloid cells [17–22], although some notable exceptions do exist [23–25]. Similarly, for gene silencing experiments, Vpx is typically delivered alongside the shRNA in VLPs to enhance knockdown efficiency. Whether Vpx has other functions in the cell or how it modulates host signaling pathways is not fully understood. Here we report that HIV-1 infection of monocytic cells with high levels of virus input can result in IFN production and ISG activation, albeit at the expense of inducing cell death. Vpx potentiates ISG responses during HIV-1 infection independently of SAMHD1 degradation, but requires the interaction with the ubiquitin-proteasome system, implicating another host factor targeted for degradation by Vpx. The ability of Vpx to increase HIV-1 infection efficiency in MDMs can be dissociated from its ability to augment ISG responses by overcoming SAMHD1 restriction in these cells. Under the circumstances where comparable infection is achieved by viruses with and without Vpx, those that contain Vpx still yield elevated responses. These results demonstrate a novel, SAMHD1-independent function of Vpx in promoting ISG expression during HIV infection, and have direct implications for studies investigating innate immune responses against retroviruses in myeloid cells.

## Results

### Vpx elevates ISRE responses in PMA-differentiated THP-1 cells during HIV-1 infection

**Fig. 1:**
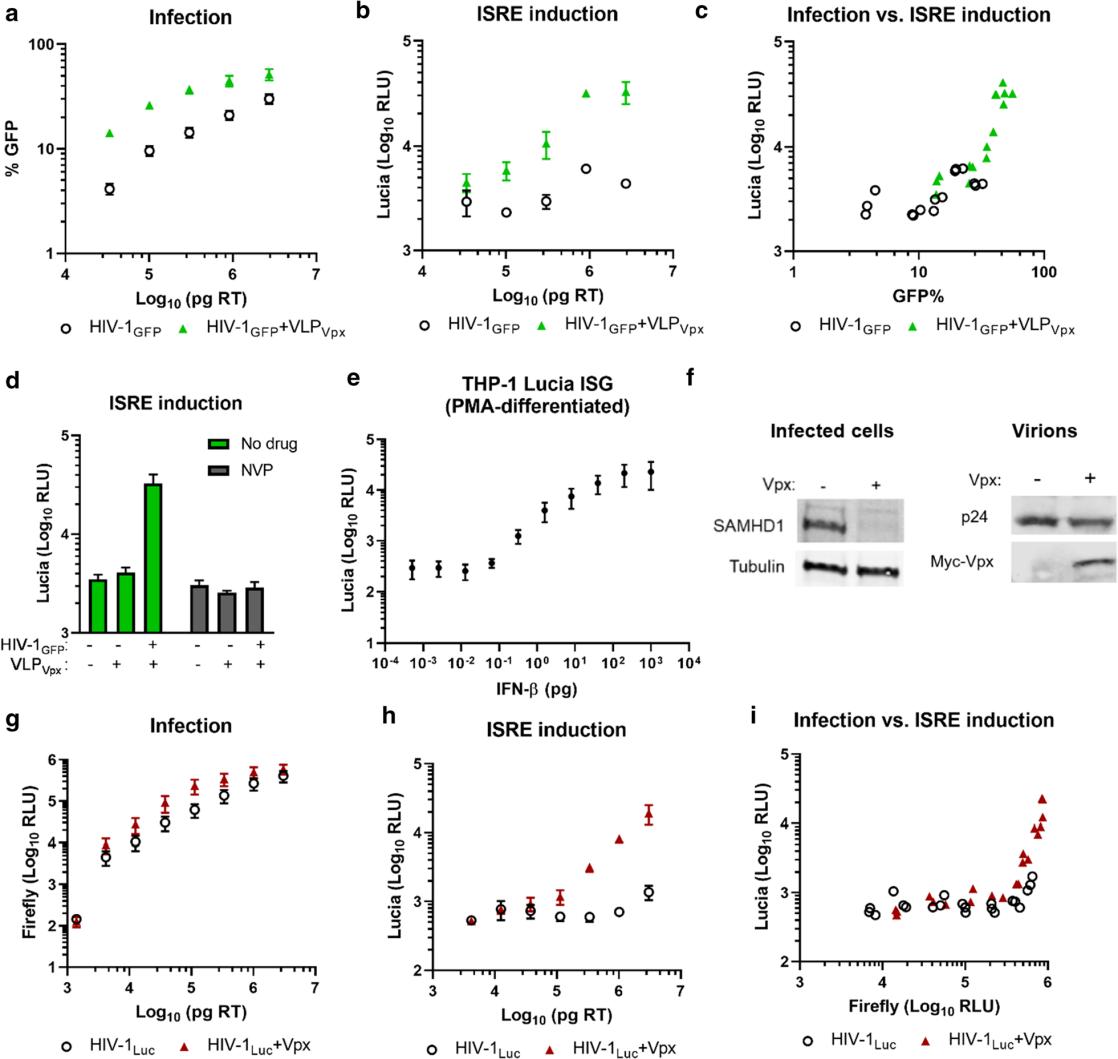
THP-1 Lucia ISG cells were differentiated overnight with PMA (25 ng/ml) and infected with serial dilutions of VSV-G pseudotyped single-round HIV-1 reporter virus in the presence of SIV_mac_ Vpx, delivered either separately as VLPs (**a**–**d**) or packaged into the virions (**g-i**). Infection levels were measured by flow cytometry (HIV-1_GFP_) or firefly luciferase assay (HIV-1_Luc_), and ISRE-driven reporter induction was quantified by lucia luciferase assay from culture supernatants 3 days post-infection. **a** Infection was measured by flow cytometry. **b** ISRE reporter induction was quantified by lucia luciferase expression. **c** Comparison of infection vs. ISRE induction. **d** Cells were transduced with VLP_Vpx_ alone or with HIV-1_GFP_ in the presence or absence of NVP; ISRE driven luciferase activity was quantified. **e** IFN-β was titrated on PMA-differentiated THP-1 Lucia ISG cells; reporter activity was measured after 3 days. **f** Western blot of total cell lysates after transduction with or without of Vpx showing SAMHD1 levels (left), and levels of p24 and Vpx-myc in virions (right). **g** Infection was quantified by firefly luciferase at 3 dpi. **h** ISRE reporter induction was quantified by lucia luciferase expression. **i** Comparison of infection levels vs. reporter induction. VLP: virus-like particle; ISRE: IFN-stimulated response element; NVP: nevirapine. Data are from three replicates; error bars depict standard deviation

To investigate the effect of Vpx on ISG responses caused by HIV-1 infection, THP-1 Lucia ISG reporter cells (hereafter “THP-1”) were used. These cells express secreted luciferase (lucia) under the control of an ISRE (IFN-stimulated response element) promoter, which serves as a convenient substitute for assessing ISG induction. PMA-differentiated cells were infected with increasing concentrations of VSV-G pseudotyped, NL4.3-based, single-round, GFP-expressing reporter virus (hereafter HIV-1_GFP_) with or without the addition of a constant level of VLPs containing SIV_mac_239 Vpx (VLP_Vpx_). To enhance the packaging of Vpx, we used a modified construct where 10 amino acids in the p6 region has been replaced with the SIV_mac_239 version, as previously described [26]. A dose-dependent increase in infection levels was observed, and infection in the presence of VLP_Vpx_ was higher than in its absence as expected, due to the restrictive activity of SAMHD1 following PMA-differentiation (Fig. 1a). ISRE-driven reporter expression was also dose-dependent, and VLP_Vpx_ addition caused up to eight-fold higher reporter induction by HIV-1_GFP_ (Fig. 1b). For easier comparison of the different infection levels with ISRE induction, each infection value (%GFP) was plotted against its own reporter activation (RLU) level, which showed consistently higher ISRE levels in the presence of VLP_Vpx_ (Fig. 1c). VLP_Vpx_ addition alone (in the absence of virus) did not result in increased reporter activity, and inhibition of reverse transcription by nevirapine treatment blocked ISRE responses (Fig. 1d). Titration of IFN-β on reporter cells revealed a dose-dependent response in reporter activity (Fig. 1e). SAMHD1 degradation in cells receiving Vpx was observed, supporting the functionality of the packaged Vpx (Fig. 1f).

Separate VLP delivery means a higher amount of viral capsid entering the cells, which has been proposed to stimulate innate immune signaling [18]. To control for this possibility, we conducted the same experiments with a similar virus expressing firefly luciferase (HIV-1_Luc_) either alone or with Vpx packaged directly into the virions (HIV-1_Luc_ + Vpx). In line with previous results, infection of PMA-differentiated THP-1 cells was dose dependent and more efficient in the presence of virion-packaged Vpx, although this difference could be mostly overcome at higher concentrations (Fig. 1g). At equal virus doses, ISRE reporter induction reached 5–14-fold higher levels in the presence of Vpx than in its absence (Fig. 1h). Plotting infection (Firefly) against ISRE induction (Lucia) revealed higher reporter activation at comparable infection levels (Fig. 1i). These results indicate that Vpx causes enhanced ISRE activation during HIV-1 infection in PMA-differentiated THP-1 cells, even when taking into consideration the difference in infection levels.

### Vpx augments innate immune activation in undifferentiated THP-1 cells during infection

**Fig. 2:**
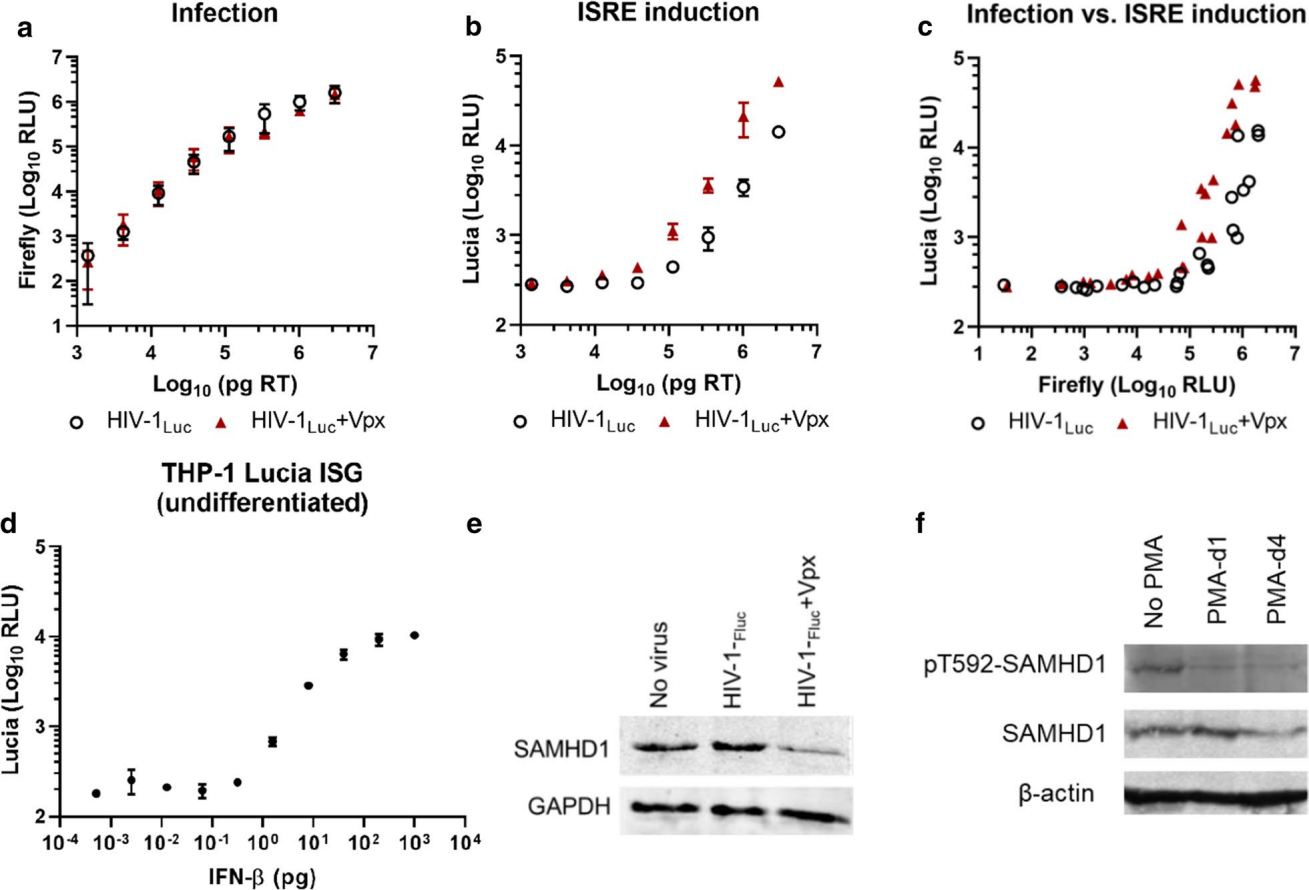
THP-1 Lucia ISG cells (undifferentiated) were infected with serial dilutions of VSV-G pseudotyped HIV-1_Luc_ reporter viruses with or without packaged SIV_mac_ Vpx. **a** Infection was quantified by firefly luciferase expression 2 days later. **b** ISRE reporter was quantified by lucia luciferase expression. **c** Comparison of infection vs. ISRE levels. **d** IFN-β was titrated on undifferentiated THP-1 reporter cells and luciferase activity in culture supernatants was measured. **e** Total cell lysates were analyzed by Western blot and probed with the indicated antibodies. **f** Representative Western blot of undifferentiated and PMA-differentiated cells (days 1 and 4 after PMA-treatment), showing phospho-T592 and total SAMHD1 levels. Data are from three replicates; error bars depict standard deviation

To overcome the difference in infection levels between viruses with or without Vpx, from here on we used undifferentiated THP-1 cells, where SAMHD1 is inactive due to the phosphorylation of a key residue [27]. Challenging undifferentiated cells with equal doses of HIV-1_Luc_ with or without virion-packaged Vpx led to equal infection levels as expected (Fig. 2a), whereas ISRE induction was still potentiated in Vpx-containing virions, on average 4.3-fold (Fig. 2b). Plotting infection against reporter induction supported these findings (Fig. 2c). Titration of IFN-β on undifferentiated reporter cells also resulted in dose-dependent reporter activation, as expected (Fig. 2d). Degradation of SAMHD1 was observed in infected cells (Fig. 2e). The loss of SAMHD1 phosphorylation in THP-1 Lucia cells following PMA differentiation after 1 and 4 days was also confirmed (Fig. 2f). These results indicate that at the same level of HIV-1 infection, the presence of Vpx causes enhanced ISG responses in undifferentiated cells where SAMHD1 is inactive.

### Virus input necessary for stimulating innate immunity causes high levels of cell death

**Fig. 3:**
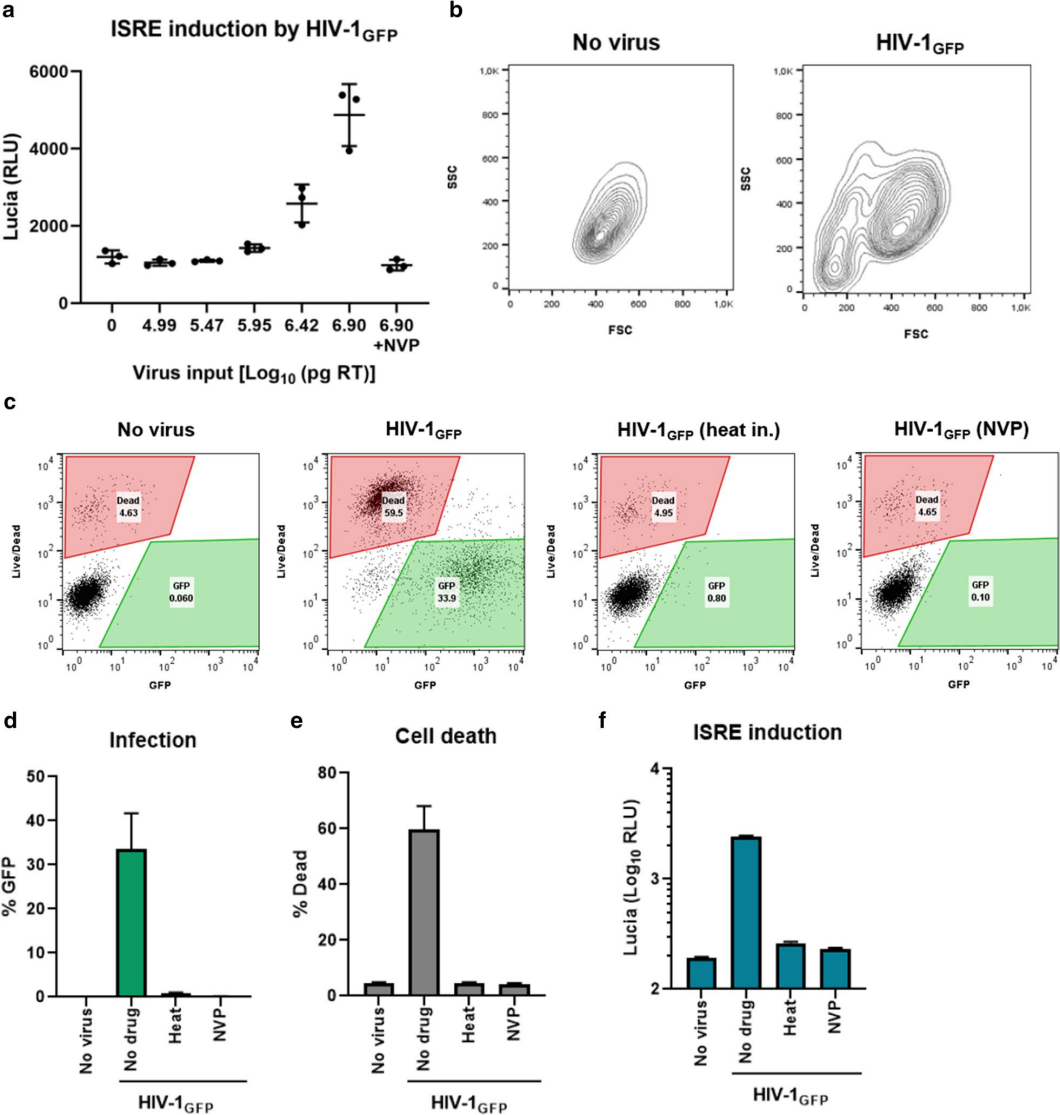
**a** THP-1 Lucia ISG cells (undifferentiated) were infected with serial dilutions of VSV-G pseudotyped HIV-1_GFP_ reporter viruses; nevirapine (NVP; 10 µM) was added as a control. ISRE reporter was quantified by lucia luciferase expression 2 days later. **b** Cells infected at the highest dose were analyzed by flow cytometry showing the shift in FSC/SSC profiles. **c** Live/dead staining vs. GFP expression confirmed cell death. **d**–**f** cells were challenged with HIV-1_GFP_ in the presence or absence of NVP (10 µM) or with heat inactivated virus. Infection efficiency (**d**) and cell death (**e**) were evaluated by flow cytometry, ISRE reporter activity was quantified by luciferase assay of culture supernatants (**f**)

We and others previously reported that HIV-1 does not strongly induce innate immune responses, unless the virus is supplemented with accessory genes from other lentiviruses, carries structural or enzymatic mutations, or is treated with chemicals that alter its uncoating kinetics or interfere with its engagement with host factors [23, 24, 28–30]. We present here that ISG induction can occur when cells are challenged with excessively high virus concentrations (Fig. 3a). Based on the estimation that one viral core contains ~ 1500 CA monomers, p24 quantification of viruses revealed that the two highest input doses correspond to 1.3 × 10^10^ to 4 × 10^10^ viral particles [31–33]. Importantly, at the highest dose presented here (8 × 10^6^ pg RT units), flow cytometry profiles of infected cells revealed a drastic shift in the size and complexity (FSC vs. SSC) compared to uninfected cells, suggesting a substantial level of cell death (Fig. 3b). We repeated the infection with the same virus input level, this time with heat inactivated virus or in the presence of 10 µM NVP. Live/dead staining confirmed that this shift was caused by extensive death (45–60%) in cells challenged with virus, compared to the basal rate of cell death (3–5%) in uninfected cells (Fig. 3c). GFP+ and dead cells formed two separate populations, suggesting that it is not the productively infected cells that are dying, although it remains possible that the GFP signal would be lost upon death. The pathway of cell death and the details of how it occurs are beyond the scope of this study. However, it should be mentioned that heat inactivation or RT inhibition prevented infection, cell death, and ISG activation (Fig. 3d–f), indicating that death was not caused by impurities in the viral supernatants and required at least the initiation of reverse transcription. Notably, the ISRE induction was not dissociable from cell death, as treatments that blocked ISRE induction also reversed the death phenotype. These results indicate that bombarding the cells with excessive amounts of virus can result in innate immune induction, however it comes at the cost of inducing massive amounts of cell death in this cell type.

### The activity of Vpx in enhancing ISG responses occurs in the absence of SAMHD1

**Fig. 4:**
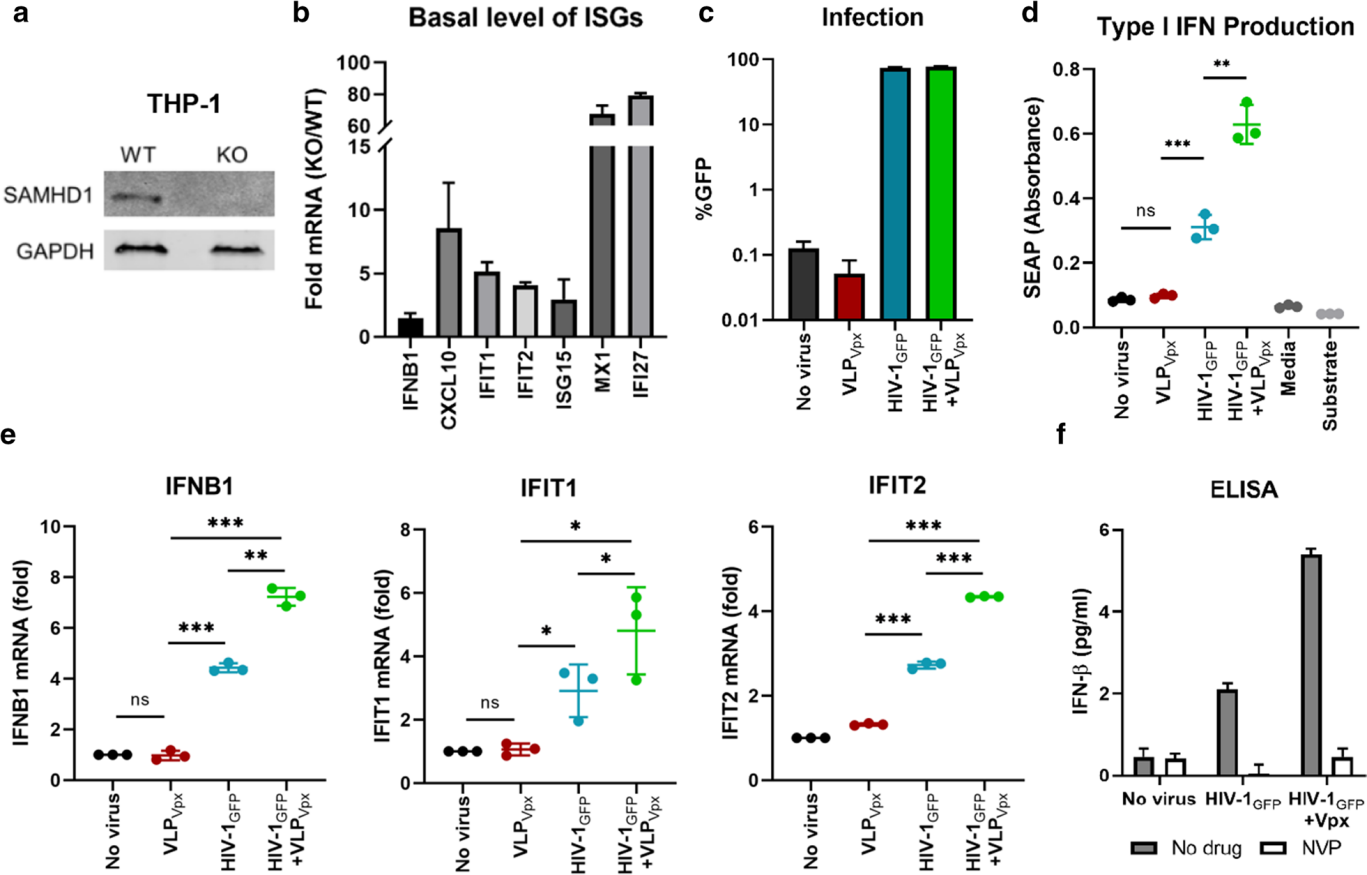
SAMHD1 KO THP-1 cells (undifferentiated) were infected with HIV-1_GFP_ in the presence or absence of VLPs containing Vpx. **a** Lysates from WT and KO cells were analyzed for SAMHD1 by Western blot. **b** Differences in basal mRNA levels between WT and KO cells for a panel of ISGs. **c** Infection levels were analyzed by flow cytometry. **d** Supernatants from infected cells were incubated with HEK-Blue IFN-α/β cells (HEK-Blue); SEAP assay was performed the next day to assess the presence of type I IFN in culture supernatants of infected cells. **e** RNA was isolated from infected cells and qRT-PCR was performed to quantify the mRNA levels of *IFNB1*, *IFIT1* and *IFIT2* (relative to *HPRT1*), and normalized to uninfected cells. **f** SAMHD1 KO THP-1 cells (undifferentiated) were infected HIV-1_GFP_ with or without virion packaged Vpx, in the presence and absence of 10 µM NVP. Supernatants were analyzed for IFN-β secretion by ELISA. *p < 0.05, **p < 0.01, ***p < 0.001, ns: not significant

To investigate whether the ability of Vpx to enhance innate immune activation during HIV-1 infection was linked to SAMHD1, we assayed infection and ISG induction in SAMHD1 KO cells (Fig. 4a). The basal mRNA expression of innate immunity-related genes was higher in SAMHD1 KO cells compared to WT controls, and ranged from 2–8-fold for *IFNB1*, *IFIT1*, *IFIT2*, *ISG15*, *CXCL10* and 60–70-fold for *MX1* and *IFI27* (Fig. 4b). Challenging cells with the same amount of HIV-1_GFP_ yielded the same level of infection (74–78%) regardless of VLP_Vpx_ addition (Fig. 4c). To compare the relative amount of type I IFN production, supernatants were collected from infected cells and assayed on HEK-Blue IFN-α/β reporter cells (hereafter HEK-Blue), which allows the detection of bioactive type I IFN by secreted alkaline phosphatase (SEAP) that can be quantified by a colorimetric assay. Supernatants from SAMHD1 KO cells that were uninfected or infected only with VLP_Vpx_ did not yield any reporter activity over the baseline, indicating a lack of type I IFN production (Fig. 4d). HIV-1_GFP_ infection resulted in marked induction of SEAP activity, which was significantly higher in the presence of VLP_Vpx_ than in its absence, despite similar infection levels (Fig. 4d). The mRNA levels for *IFNB1*, *IFIT1* and *IFIT2* followed the same pattern as type I IFN production (Fig. 4e). To quantify directly the amount of type I IFN produced, SAMHD1 KO cells were infected with HIV-1_GFP_ with or without virion packaged Vpx, in the presence or absence of NVP, and IFN-β was quantified from culture supernatants 3 days later. Low but detectable level of IFN-β was produced in response to infection with HIV-1_GFP_, which was potentiated in the presence of Vpx (Fig. 4f). In both cases, NVP treatment reversed the IFN-β production. Taken together, these results demonstrate that addition of Vpx during HIV-1 infection increases type I IFN and ISRE induction even in the absence of SAMHD1.

### Vpx enhances innate immune responses to HIV-1 by targeting a host factor for degradation

**Fig. 5:**
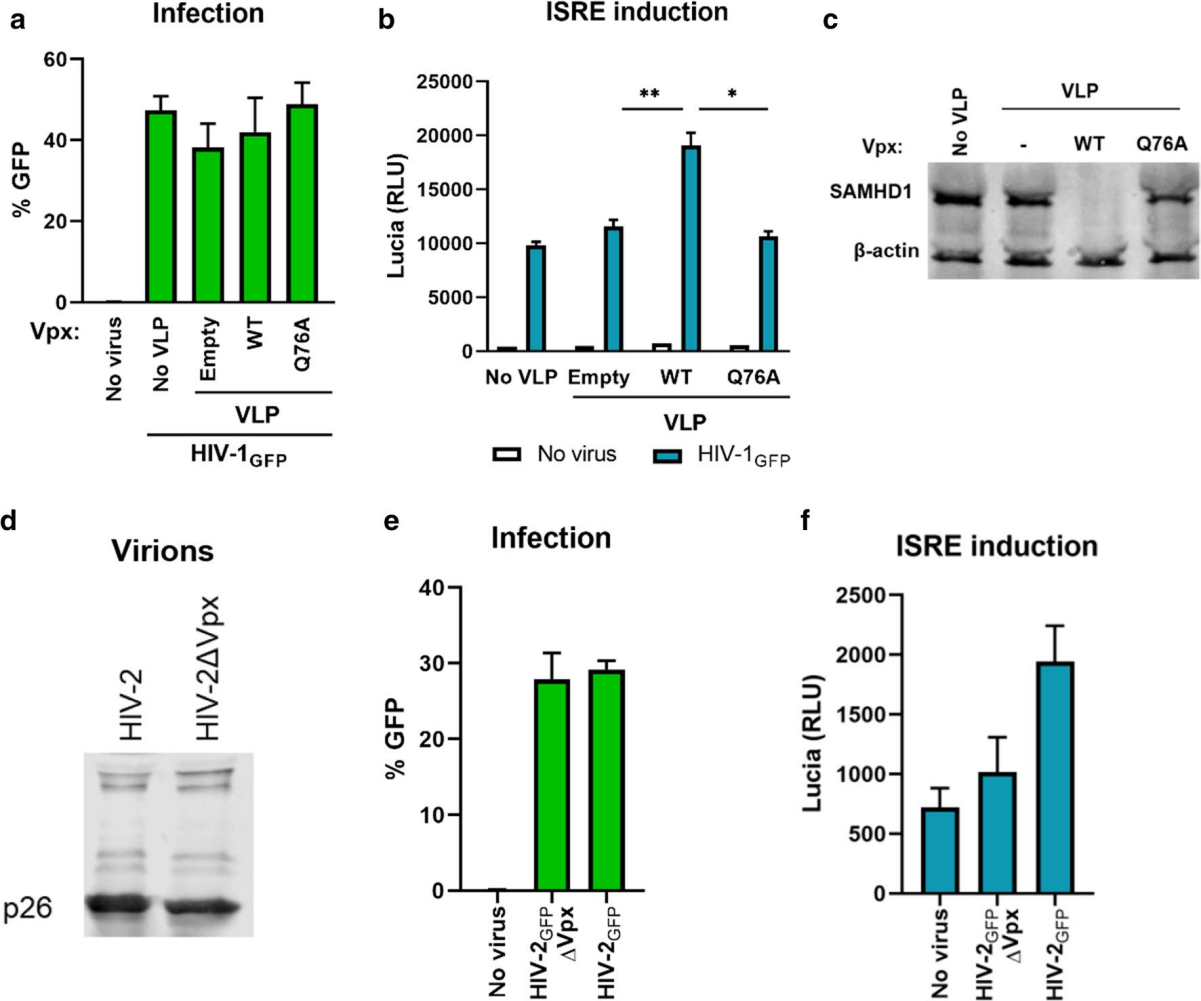
**a**–**c** THP-1 Lucia ISG cells (undifferentiated) were infected with HIV-1_GFP_ in the presence or absence of VLPs containing WT or Q76A Vpx. **a** Infected cells were analyzed by flow cytometry 2 days post-infection. **b** ISRE reporter induction was assessed by lucia luciferase assay of supernatants from infected cells. **c** Cell lysates were collected from infected cells and analyzed on a Western blot, probing for SAMHD1. **d**–**f** THP-1 Lucia ISG cells (undifferentiated) were infected with a single-round HIV-2_GFP_ with or without its own Vpx. **d** Virions were run on a Western blot and probed for p26 CA using a p24 antibody known to cross react with HIV-2 CA [47]. Infection was quantified by flow cytometry (**e**) and ISRE induction was measured by luciferase activity (**f**). *p < 0.05, **p < 0.01

Vpx can target a number of host proteins for degradation through its interaction with DCAF1 in infected cells. However, it was shown that Vpx can also affect certain host processes independently of its ability to degrade host proteins, for instance via STING-mediated activation of the NF-κB pathway [34]. To investigate whether the enhanced immune responses by Vpx is caused by its ability to target proteins for degradation, VLPs containing Q76A mutant of Vpx were generated, which is defective in DCAF1 binding. Undifferentiated THP-1 reporter cells were infected with HIV-1_GFP_ in the presence or absence VLPs that were either empty, or containing WT or Q76A Vpx. Infection levels were similar (~ 50%) in all cases, irrespective of the VLP presence or their contents (Fig. 5a). ISRE reporter activity was significantly higher in the presence of WT Vpx, whereas Q76A mutation abolished this response (Fig. 5b). WT Vpx, but not the Q76A mutant, efficiently degraded SAMHD1 in infected cells (Fig. 5c). Thus, the ability of Vpx to interact with the ubiquitin-proteasome system to target proteins for degradation is required for this phenotype.

### The enhancement of ISRE response is conserved in HIV-2 Vpx

We next asked whether HIV-2, which contains a *vpx* gene, mirrors the phenotype seen with SIV_mac_ Vpx. We infected THP-1 reporter cells with a single-round, VSV-G pseudotyped HIV-2_GFP_ that either carried *vpx* or not. Using viruses with similar levels of p26 capsid (Fig. 5d), we observed similar infection levels in undifferentiated cells, as expected (Fig. 5e), but higher ISRE reporter activation in the presence of HIV-2 Vpx (Fig. 5f). These results suggest that the increased innate immune activation phenotype of SIV_mac_ Vpx is also conserved in HIV-2 Vpx.

### A combined approach to overcome SAMHD1 restriction exposes an innate immune enhancement activity of Vpx

To test whether the increased innate immune responses during HIV-1 infection by Vpx also occurs in primary cells, we infected monocyte-derived macrophages (MDMs) with HIV-1_GFP_ with or without packaged Vpx. Macrophages are largely refractory to infection by HIV-1 in the absence of Vpx due to the activity of SAMHD1. At equal input of Vpx− and Vpx+ viruses, a pronounced difference in infection was observed, concomitant with SAMHD1 degradation (Additional file 1: Fig. S1A, B). In accordance with the high infection level in the presence of Vpx, there was also a marked increase in the mRNA expression profiles for a panel of ISGs (Additional file 1: Fig. S1C). In an effort to overcome the infectivity difference between viruses containing and lacking Vpx, we used different input doses of HIV-1_Luc_ to reach the same level of infection, which was achieved when 500 times more Vpx− virus was used compared to the Vpx+ virus (data not shown). However, due to volume limitations, it was not possible to simply add 500-fold more virus, but rather the Vpx+ virus input had to be decreased in order to get equal infection levels. Reducing the amount of virus input, in turn, reversed any ISG induction that could previously only be observed at the highest doses of virus (Fig. 3a, and data not shown). These results revealed the challenges of dissociating the infectivity boost conferred to HIV-1 by Vpx from its enhancement of innate immune responses during MDM infection, and emphasized the necessity of overcoming SAMHD1 restriction in other ways.

**Fig. 6:**
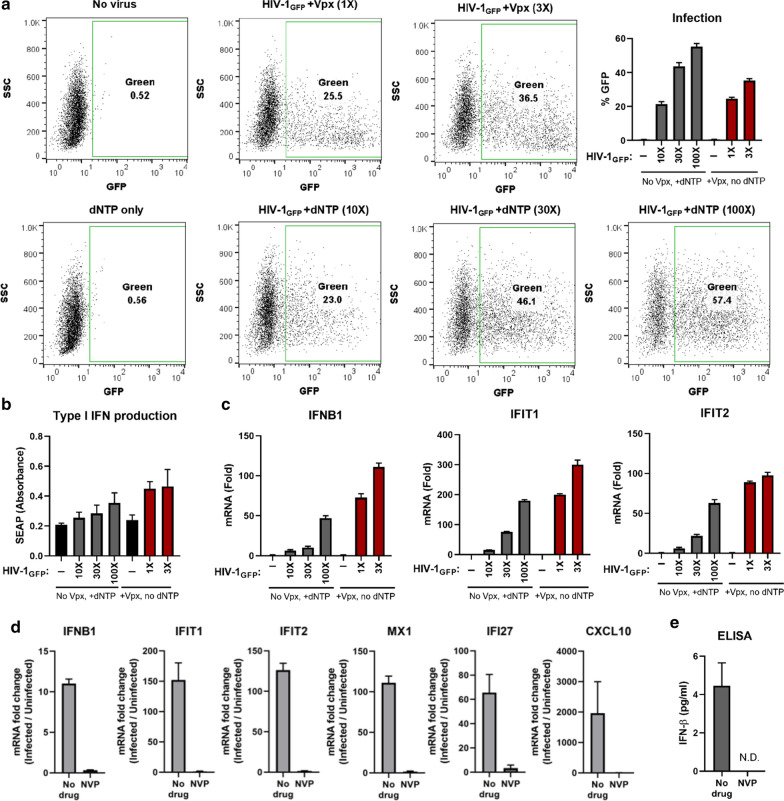
**a**, **b** MDMs were infected with different amounts of HIV-1_GFP_ with or without virion-packaged Vpx in the presence or absence of extracellular dNTP addition to match infection levels. **a** Infection was assessed by flow cytometry. **b** Supernatants from infected cells were assayed on HEK-Blue IFN-α/β cells for the presence of type I IFN. **c** mRNA levels for *IFNB1*, *IFIT1* and *IFIT2* were quantified by qRT-PCR and normalized to uninfected cells. **d** MDMs were infected with HIV-1_GFP_ with dNTP addition, in the presence or absence of 10 µM NVP. mRNA levels for the indicated ISGs were measured by RT-qPCR, normalized to *HPRT1* and then to uninfected cells. **e** Supernatants from the infected MDMs in **d** were assessed for IFN-β secretion by ELISA. N.D.: Not detected.

To equalize infection levels in MDMs between viruses containing and lacking Vpx, it was necessary to overcome SAMHD1 restriction through other means. Knockdown of SAMHD1 using siRNA showed only limited success, and addition of dNTPs partially, but not fully, overcame SAMHD1 restriction (data not shown). Using an excess of HIV-1_GFP_ (10–100-fold) simultaneously with dNTP addition resulted in comparable infection levels with its Vpx+ counterpart, allowing direct assessment of the effects the presence of Vpx might have on ISG induction (Fig. 6a). In case of HIV-1_GFP_ with excess virus input and 2.5 mM dNTPs, GFP + cells ranged from 23–57%, whereas in the case of HIV-1_GFP_ + Vpx (no dNTP) infection level was between 25–37% (Fig. 6a). Supernatants from infected cells were analyzed for type I IFN production on HEK-Blue reporter cells, where Vpx + viruses yielded a higher activity than Vpx- viruses (Fig. 6b). For all virus concentrations tested, the mRNA expression for *IFNB1*, *IFIT1* and *IFIT2* were upregulated to a higher degree in the presence of Vpx than in its absence, despite comparable infection levels (Fig.  6c; 1× vs. 10×) and in some cases even though Vpx-containing virus resulted in less GFP + cells (Fig. 6c; 3× vs. 100×). The difference between mRNA expression profiles induced by HIV-1_GFP_ and HIV-1_GFP_ + Vpx ranged from 6–47-fold vs. 73–111-fold for *IFNB1*, 16–180-fold vs. 200–300-fold for *IFIT1*, and 6–63-fold vs. 89–98-fold for *IFIT2*, respectively, in agreement with the HEK-Blue profiles. To ensure that the observed IFN response was due to successful infection, we repeated the MDM infection with HIV-1_GFP_ supplemented by dNTPs in the presence and absence of NVP. Infection resulted in increased mRNA expression of our ISG panel (Fig. 6d), as well as increased secretion of IFN-β (Fig. 6e), both of which were sensitive to RT inhibition. As mentioned above, it is rather complicated to reach exactly the same infection level in MDMs with viruses that differ in their Vpx content. Challenge with excess virus translates to an excess number of viral cores entering the cells, as viruses are VSV-G pseudotyped and no additional block is expected at the entry level. Despite the higher number of capsids entering the cells and ultimately achieving similar infection levels in MDMs, viruses containing Vpx still resulted in markedly increased ISG responses.

### Vpx mildly increases IFN-β and ISRE-driven transcriptional activity

To investigate whether Vpx enhances transcription from a type I IFN inducible promoter, we transfected HEK-Blue cells with Vpx or a control plasmid and assayed for SEAP reporter in culture supernatants. Treatment of reporter cells with IFN-β, but not IFN-γ, resulted in strong induction of SEAP activity compared to untreated controls; however, this activation was not altered by the presence of Vpx (Additional file 2: Fig. S2A). The cells responded to type I IFN treatment in a dose-dependent manner, while VLP delivery, whether empty or Vpx-containing, did not affect the reporter levels triggered by the different IFN doses (Additional file 2: Fig. S2B). To test whether Vpx alters innate immune responses in the context of a different virus that strongly activates IFN response, THP-1 cells were infected with Sendai Virus (SeV) either alone, or in combination with VLPs with or without Vpx. SeV infection resulted in strong ISRE reporter expression, but VLP_Vpx_ addition did not alter the reporter activity (Additional file 2: Fig. S2C). In each case, it is possible that IFN-β treatment or SeV infection promoted such high levels of induction that the system was saturated towards any additional effects by Vpx. To control for this possibility, we co-transfected 293T cells with transcriptional reporter constructs for IFN-β and ISRE-driven luciferase plasmids, along with Vpx or a control GFP-expressing plasmid. Reporter activation revealed a mild (3–4 fold) average stimulation of basal levels in the presence of Vpx compared to control plasmid for both IFN-β and ISRE promoters (Additional file 2: Fig. S2D). To overcome potential transfection-dependent artifacts, we repeated the reporter assays in 293T cells transduced with empty or Vpx-containing VLPs, the latter resulting in higher reporter activity from both promoters (Additional file 2: Fig. S2E). Overall these results suggest that Vpx can cause transcriptional activation of promoters relevant to innate immune signaling pathways, albeit at relatively mild levels, which may help explain the enhanced innate immune responses observed during HIV-1 infection in the presence of Vpx.

## Discussion

We show here that the presence of Vpx during HIV-1 infection, whether packaged into the virions or delivered separately as VLPs, potentiates innate immune responses in differentiated and undifferentiated THP-1 cells, as well as MDMs. This effect cannot be accounted for by SAMHD1 targeting, as SAMHD1 KO cells also show elevated responses in the presence of Vpx. Notably, a detectable ISG response is only feasible using excessive amounts of virus, at least in THP-1 cells, which ends up causing high levels of cell death independently of the presence or absence of Vpx. The DCAF1 interaction-deficient Vpx mutant fails to augment ISG expression, thus the mechanism likely involves targeting a host protein by Vpx for proteasomal degradation. Overcoming the difference in infection levels in MDMs via dNTP addition and using a high ratio of Vpx−/Vpx+ viruses, we uncover an innate immune enhancement phenotype of Vpx that occurs even in the absence of SAMHD1.

Several studies have implicated Vpx in the negative regulation of innate immune signaling pathways, specifically the NF-κB pathway. In one study, Vpx was shown to inhibit STING-induced NF-κB responses without altering STING-induced IRF3 responses [34]. Although the mechanism is not known, inhibition of NF-κB responses by Vpx required direct interaction with STING, but was independent of the ability of Vpx to target host proteins for degradation, as the Q76A mutant was still functional. In a second study, Vpx was shown to inhibit NF-kB signaling in response to all stimuli, by directly interacting with the p65 subunit, although not involving proteasomal degradation [35]. These reports describe an activity of Vpx that is different than the one described in our study, in which the Q76A mutant no longer stimulates ISG responses. Thus, the ability of Vpx to increase ISG responses most likely involves a mechanism involving ubiquitin-mediated protein degradation, although it is not possible to rule out additional effects that this mutation might have on Vpx function. HIV-1 infection was previously reported to stimulate type I IFN production through the IRF pathway [20, 36, 37], which we show is potentiated by Vpx, thus it remains possible that NF-κB responses are impaired in the presence of infection with Vpx, whereas the IRF3/IRF7 signaling remains active.

SAMHD1 was shown to be critical for the replication stress response, where it helps remove DNA fragments released from stalled replication forks [38]. In the absence of SAMHD1, constitutive activation of the cGAS/STING pathway ensues, providing an explanation as to why mutations in this locus is linked to the development of various cancers and the autoimmune disease Aicardi-Goutières Syndrome. In addition, SAMHD1 was shown to inhibit NF-κB responses by interacting with the p50 and p52 subunits, and to block IRF7-mediated ISRE transactivation [39]. Given its role in counteracting intrinsic immune pathways, it is perhaps not surprising that targeting SAMHD1 for proteasomal degradation results in the activation of these pathways ordinarily kept in check. An interesting finding of this study is that Vpx has the ability to trigger ISRE activation during HIV-1 infection to higher levels even in the absence of SAMHD1, indicating the involvement of a separate mechanism. The exact determinants of this activation remain to be elucidated, but may involve increased transcriptional activity from IFN-β and ISRE-responsive promoters.

In reporter assays using 293T cells, we observed only a mild increase in transcriptional activity from the IFN-β and ISRE-driven expression. Whether this relatively modest stimulation is sufficient to explain the elevated responses observed during HIV-1 infection remains under discussion. As mentioned above, loss of SAMHD1 can trigger the activation of antiviral signaling pathways, suggesting Vpx may have both SAMHD1-dependent and independent functions with regard to stimulation of innate immune responses during infection. Interestingly, the presence of Vpx during infection of THP-1 cells with Sendai Virus did not cause a significant difference in ISG stimulation, indicating Vpx may have a specific role of stimulating responses during HIV-2 or SIV infections, possibly through its interaction with viral proteins or with host proteins activated during infection. Alternatively, the strong IFN response caused by SeV infection could potentially mask any modest effects that Vpx may exert during infection.

The *vpx* gene is homologous to the *vpr* gene present in all primate lentiviruses. As HIV-1 does not contain Vpx or any other gene that counteracts the function of SAMHD1, the significance of this restriction factor for HIV-1 replication in vivo has remained uncertain. In a pig-tailed macaque model of SIVsmm infection, deletion of *vpx* resulted in a significant defect in replication [40]. Moreover, in simultaneous infection of WT and Δ*vpx* viruses, the mutant virus is readily outcompeted by the WT virus, underlining the importance of this viral gene in vivo [40]. SIVmne infection of pig-tailed macaques with Δ*vpx* virus resulted in significantly lower levels of plasma viremia [41]. Therefore, Vpx seems to be necessary for viral replication in vivo, and enhanced ISG responses could pose a conflict for the virus, which largely goes “under the radar” compared to other viruses. From an evolutionary standpoint it may seem counterintuitive for a virus to keep a gene that causes the activation of host antiviral pathways. We reason that it is not a primary function of Vpx, but rather a side-effect that needs to be tolerated for efficient infection. Enhanced activation of ISG responses might be a small compromise in exchange for the significant improvement of infection levels for SIVs and HIV-2 in vivo. Although the stimulation by Vpx occurs in a SAMHD1-independent manner, this type of compromise could be at play that balances an activity of Vpx that is beneficial to the virus, while enhancing innate immune responses.

## Conclusions

Retroviral Vpx gene from HIV-2 and some SIV strains carry out various functions during infection, including counteraction of host restriction and resistance factors. Vpx enhances the ISG signaling observed during HIV-1 infection in monocytic cell lines and primary MDMs. At equal infection levels, Vpx-containing viruses cause significantly higher ISG induction than their Vpx-lacking counterparts, even in the absence of SAMHD1. Overcoming SAMHD1 restriction in MDMs to achieve equal infection levels uncover an innate immune signaling enhancer activity of Vpx, which likely depends on the targeting of a host protein for degradation. Overall these data reveal a novel activity of Vpx and has implications for studies investigating innate immune responses against retroviral infections.

## Methods

### Cell culture

293T and HEK-Blue IFN-α/β cells (Invivogen) were maintained in DMEM containing 10% FBS and 100 µg/ml Pen/Strep (ThermoFisher). THP-1 Lucia ISG cells were from Invivogen, THP-1 SAMHD1 KO and WT control cells were kindly provided by Torsten Schaller. All THP-1 based cells were maintained in RPMI (Gibco) with 10% FBS, 100 µg/ml Pen/Strep, 100 µg/ml Normocin. For differentiation, THP-1 cells were treated with PMA (25 ng/ml) for 24 h, followed by fresh media addition. PBMCs were isolated from buffy coats of anonymous blood donors from the Red Cross using standard Ficoll separation. Monocytes were selected by adhering PBMCs in RPMI with 5% human serum, 1 mM HEPES and 24 µg/ml gentamicin for several hours, followed by washing to remove unbound cells. Monocyte-derived macrophages were differentiated from monocytes by adding GM-CSF (50 ng/ml) for 7–14 days with freshly added GM-CSF every other day.

### Virus production and infection

Virus stocks were produced by standard transfection of 293T cells with polyethylenimine (PEI), followed by media change, filtering, and ultracentrifugation with a sucrose cushion to concentrate virus, which was aliquoted and frozen at − 80 °C. Virus-containing supernatants were treated with benzonase prior to addition onto cells. Infections were done by spinoculation at 1200×*g* for 1 h at 25 °C. Infectious units were determined on TZM-Bl cells followed by X-gal staining, or in case of GFP viruses on 293T cells by flow cytometry. p24 levels were quantified by ELISA; RT activity was determined by a Taqman qPCR-based PERT assay.

HIV-1_GFP_ or HIV-1_Luc_ was produced transfection of the pNL4.3 e- r- based plasmids that carry either GFP or firefly Luciferase in place of the env gene, together with a plasmid encoding myc-tagged VSV-G env (pVSV-G). For virus packaging of Vpx, a plasmid encoding myc-tagged SIV_mac239_ *vpx* gene was transfected simultaneously. To allow efficient Vpx incorporation, pNL4.3 e- r-plasmids with a modified p6 region from SIV (amino acids 17–26) was used, as previously described [26]. HIV-2 was produced by transfection of the single-round vector pHIV2.GFP e- r- [42] or pHIV2.GFP e- r- x- (Δ*vpx*) with VSV-G plasmid. VLPs were produced by co-transfection of plasmids carrying HIV-1 gag pol with the same p6 aa 17–26 modification (pMDL gag/pol SIVp6 17–26), rev (pRSV-rev), VSV-G with or without Vpx. As a general rule, infection of difficult-to-infect cells (MDM, differentiated THP-1) was scored at 3 dpi, whereas infection of cells in which infection proceeds faster (e.g. undifferentiated THP-1) was scored at 2 dpi. ISG mRNA quantification and WB for SAMHD1 degradation were performed at 2 dpi.

### PERT assay and RT-qPCR

RT activity of viral stocks was measured by PERT assay, as previously described [43]. Briefly viral supernatants were mixed with an equal volume of lysis buffer (0.25 % Triton X-100, 50 mM KCL, 100 mM TrisHCL pH 7.4, 40 % glycerol, 0.4U/µl RNase inhibitor), incubated for 15 mins at room temperature and diluted 1:10 with water. qPCR was performed on diluted virus lysate or RT standards (Cavidi) in a 20 µl reaction containing 500 nM each of forward and reverse primers, 250 nM of the Taqman probe, 40 ng MS2 RNA, and 1X SensiFAST Probe No-ROX Master Mix (BioCat). Reactions were run on a BioRad CFX96 cycler.

For mRNA expression analysis, total RNA was collected isolated using RNeasy Mini Kit (Qiagen), cDNA was synthesized using Superscript III cDNA synthesis kit (Invitrogen), treated with Turbo DNase and inactivation reagent (Ambion), and qPCR was performed with the SensiFAST Probe No-ROX or SYBR Master Mix (BioCat), based on the Taqman or SYBR qPCRs. A list of primer/probe sequences and sources is provided in Table 1.

**Table 1:** List of primers and probes used in this study

Primer/Probe	Sequence (5′−3′)	References
PERT-F	TCCTGCTCAACTTCCTGTCGAG	Horie et al. [43]
PERT-R	CACAGGTCAAACCTCCTAGGAATG	
PERT-Probe	TCTTTAGCGAGACGCTACCATGGCTA	
IFNB1-F	TTGACATCCCTGAGGAGATTAAGC	Hillyer et al. [44]
IFNB1-R	TTAGCCAGGAGGTTCTCAACAATAG	
IFNB1-Probe	CCAGAAGGAGGACGCCGCATTGACC	
HRPT1-F	TCTTTGCTGACCTGCTGGATT	This paper
HPRT1-R	TTATGTCCCCTGTTGACTGGT	
HPRT1-probe	AGTGATAGATCCATTCCTATGACTGT	
IFIT1-F	AACAGGTTTTCGCAATCAGGC	
IFIT1-R	GCTCCAGACTATCCTTGACCTG	
IFIT1-probe	AGATTGCCTCCTCCCTGGAA	
IFIT2-F	AACAGCTGAGAATTGCACTGC	
IFIT2-R	GCCAGTAGGTTGCACATTGTG	
IFIT2-probe	TTCCTTGGAGAGCAGCCTAC	
MX1-F	ATCCTGGGATTTTGGGGCTT	Sumner et al. [28]
MX1-R	CCGCTTGTCGCTGGTGTCG	
CXCL10-F	TGGCATTCAAGGAGTACCTC	
CXCL10-R	TTGTAGCAATGATCTCAACACG	
IFI44L-F	GTATAGCATATGTGGCCTTGCTTACT	Zahoor et al. [45]
IFI44L-R	ATGACCCGGCTTTGAGAAGTC	
IFI27-F	GGCAGCCTTGTGGCTACTCT	
IFI27-R	ATGGAGCCCAGGATGAACTTG	
ISG15-F	ACTCATCTTTGCCAGTACAGGAG	
ISG15-R	CAGCATCTTCACCGTCAGGTC	

Primer/Probe sets	Catalog number	Company
IFNB1	Hs01077958_s1	ThermoFisher
CXCL10	Hs00171042_m1	
IFIT1	Hs01675197_m1	
IFIT2	Hs00533665_m1	
HPRT1	Hs02800695_m1	
GAPDH	Hs02786624_g1	

### Luciferase assays

IFN-β-Luc and ISRE-Luc plasmids were described before [46]. For firefly luciferase, cells were lysed in passive lysis buffer and mixed with the assay substrate (Promega). For Lucia luciferase assays, supernatants were collected and mixed with Quanti-Luc substrate (Invivogen). Luminescence measurements were done using a LUMIstar OMEGA plate reader (BMG).

### Flow cytometry

Adherent cells were incubated with Accutase (Stemcell) at 37 °C for 20 min, or suspension cells were simply collected, washed once with PBS, stained with a fixable-viability dye (FVD-660; eBioscience), fixed in IC fixation buffer (eBioscience), and diluted in PBS to be analyzed by flow cytometry (FACScalibur, BD). Further analyses were performed in FlowJo.

### Western blot

Cells were washed with PBS and lysed in 100 mM Tris, 30 mM NaCl, 0.5% NP40. Lysates were supplemented with 4x loading buffer (Invitrogen) and ran on denaturing SDS-PAGE, transferred to PVDF membrane, incubated first with blocking solution (Rockland), then with primary antibodies (1:1000), washed with PBS-T, incubated with IRdye-labeled secondary antibody (Licor), washed, and scanned using Odyssey infrared scanner (Licor). The primary antibodies used were: mouse anti-SAMHD1 (Bio-Rad), rabbit anti-pT592-SAMDH1 (Cell Signaling Technology), mouse anti-tubulin (Sigma); mouse anti-GAPDH (Thermo), mouse anti-myc (Sigma).

### SEAP assay and ELISA

Supernatants to be assayed were collected from infected cells and incubated in 96-well plates (25K/well) with freshly split HEK-Blue IFN-α/β cells overnight. The next day, supernatants from HEK-Blue cells were mixed with Quanti-Blue reagent (Invivogen) and absorbance was measured at 650 nm. Type I IFN secretion in culture supernatants was quantified by the IFN-α “all subtype” and IFN-β ELISA kits according to manufacturer’s instructions (PBL).

## Supplementary Information

**Additional file 1: Figure S1.** (A-C) MDMs were infected with equal amounts of HIV-1_GFP_ with or without virion-packaged Vpx, in the presence or absence of 10 µM NVP. Infection levels were analyzed by flow cytometry (representative flow cytometry profiles) (A) and SAMHD1 degradation was assayed by Western blot (B). mRNA levels for a panel of ISGs were quantified by qRT-PCR, normalized to *HPRT1* and to uninfected cells (C).

**Additional file 2: Figure S2.** (A) HEK-Blue IFN-α/β SEAP reporter cells were transfected with Vpx or a GFP plasmid as a control, and stimulated or not with IFN-γ or IFN-β. Secreted alkaline phosphatase activity was measured 1 day later in culture supernatants. (B) HEK-Blue IFN-α/β SEAP reporter cells were left untreated, or transduced with empty VLPs or VLP_Vpx_, and treated with different concentrations of IFN-β (0.01-1 ng) one day later. SEAP activity in culture supernatants was measured 1 day after IFN-β addition. (C) THP-1 Lucia ISG cells (undifferentiated) were treated with VLPs that are either empty or containing Vpx and infected with Sendai Virus. One day after infection, reporter activity in the supernatants was measured by luciferase assay. (D) 293T cells were co-transfected with luciferase reporter constructs plus Vpx or a control plasmid. Reporter activity (firefly) was measured in cell lysates 1 day after transfection, values are given as fold induction of Vpx over control. (E) Experiment was performed as in panel D, except cells were transduced with empty or Vpx-containing VLPs prior to transfection with reporter constructs. ****p < 0.0001, ns: not significant.

## Data Availability

The datasets used and/or analyzed during the current study are available from the corresponding author on reasonable request.

## Abbreviations

HIV-1/HIV-2: Human immunodeficiency virus 1 and 2
SIV: Simian immunodeficiency virus
IFN: Interferon
ISG: Interferon-stimulated gene
ISRE: Interferon-stimulated response element
SEAP: Secreted alkaline phosphatase
PERT: Product enhanced reverse transcriptase
SAMHD1: SAM and HD domain containing deoxynucleoside triphosphate triphosphohydrolase 1
NVP: Nevirapine
RLT: Raltegravir
RT: Reverse transcriptase
IN: Integrase.
CA: Capsid
RLU: Relative light units
MDM: Monocyte-derived macrophage
Vpx: Viral protein X
