# Supplementary figures and images for: Vpx enhances innate immune responses independently of SAMHD1 during HIV-1 infection
Source: Retrovirology. 2021 Feb 9;18:4. doi: 10.1186/s12977-021-00548-2 (PMC7871410; doi:10.1186/s12977-021-00548-2)

Figure S1.

Cingöz *et al.*

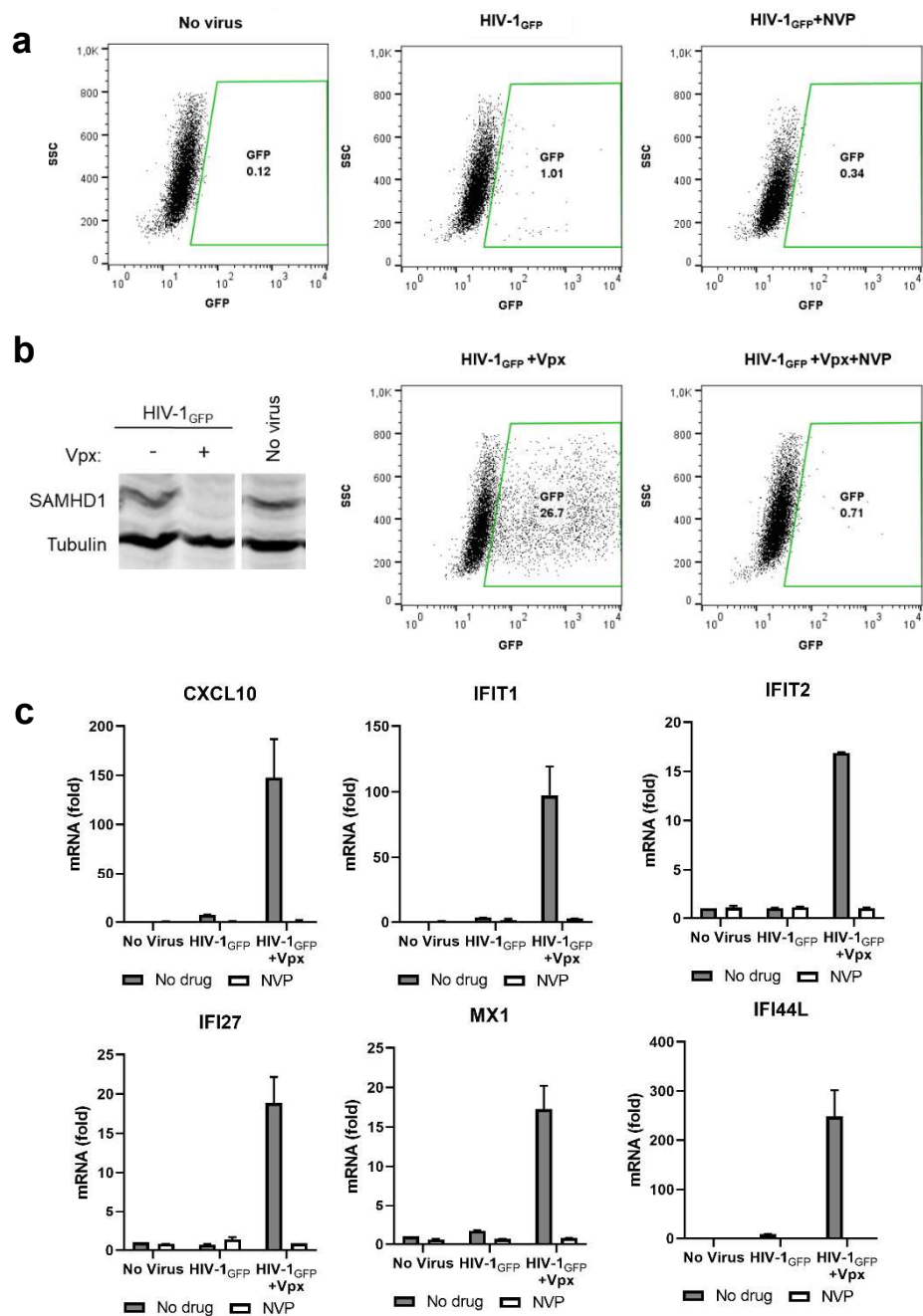

Supplement: Supplementary file 1 — Additional file 1: Figure S1. (A-C) MDMs were infected with equal amounts of HIV-1GFP with or without virion-packaged Vpx, in the presence or absence of 10 µM NVP. Infection levels were analyzed by flow cytometry (representative flow cytometry profiles) (A) and SAMHD1 degradation was assayed by Western blot (B). mRNA levels for a panel of ISGs were quantified by qRT-PCR, normalized to HPRT1 and to uninfected cells (C). [file 12977_2021_548_MOESM1_ESM.pdf]

Figure S2.

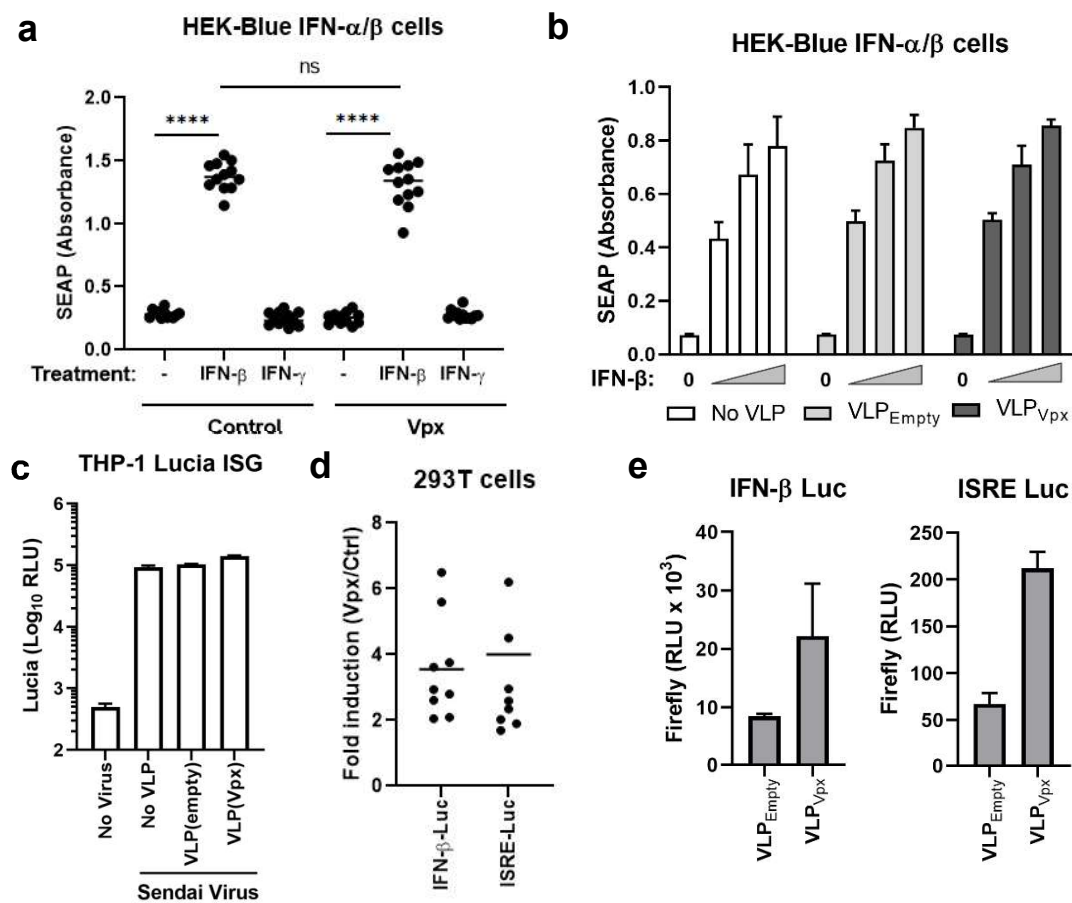

Supplement: Supplementary file 2 — Additional file 2: Figure S2. (A) HEK-Blue IFN-α/β SEAP reporter cells were transfected with Vpx or a GFP plasmid as a control, and stimulated or not with IFN-γ or IFN-β. Secreted alkaline phosphatase activity was measured 1 day later in culture supernatants. (B) HEK-Blue IFN-α/β SEAP reporter cells were left untreated, or transduced with empty VLPs or VLPVpx, and treated with different concentrations of IFN-β (0.01-1 ng) one day later. SEAP activity in culture supernatants was measured 1 day after IFN-β addition. (C) THP-1 Lucia ISG cells (undifferentiated) were treated with VLPs that are either empty or containing Vpx and infected with Sendai Virus. One day after infection, reporter activity in the supernatants was measured by luciferase assay. (D) 293T cells were co-transfected with luciferase reporter constructs plus Vpx or a control plasmid. Reporter activity (firefly) was measured in cell lysates 1 day after transfection, values are given as fold induction of Vpx over control. (E) Experiment was performed as in panel D, except cells were transduced with empty or Vpx-containing VLPs prior to transfection with reporter constructs. ****p < 0.0001, ns: not significant. [file 12977_2021_548_MOESM2_ESM.pdf]
